# The Ratio of Anterior and Posterior Vertebral Heights Reinforces the Utility of DXA in Assessment of Vertebrae Strength

**DOI:** 10.1007/s00223-014-9868-1

**Published:** 2014-05-23

**Authors:** Grzegorz Tatoń, Eugeniusz Rokita, Mariusz Korkosz, Andrzej Wróbel

**Affiliations:** 1 Department of Biophysics, Jagiellonian University Medical College, Łazarza 16, 31530 Kraków, Poland; 2 Division of Rheumatology, Department of Internal Medicine and Gerontology, Jagiellonian University Medical College, Śniadeckich 10, 31-531 Kraków, Poland; 3 Institute of Physics, Jagiellonian University, Reymonta 4, 30-059 Kraków, Poland

**Keywords:** BMD, DXA, Vertebral strength, Bone fracture risk

## Abstract

The objective of the study was to introduce a new parameter describing bone strength with greater precision than the widely used antero-posterior DXA (dual-energy X-ray absorptiometry), which measures areal bone mineral density (aBMD). The adjusted areal bone mineral density (AaBMD) defined as the ratio between aBMD and *h*_a_/*h*_p_ (*h*_a_ and *h*_p_: anterior and posterior vertebral body heights measured on the lateral view, respectively) is proposed: AaBMD = aBMD/(*h*_a_/*h*_p_). The utility of AaBMD in prediction of bone strength was assessed by in vitro measurements of cadaver L3 vertebrae. The AaBMD of 31 vertebrae was correlated with the ultimate stress (*P*_max_) and load (*F*_max_) values obtained in mechanical tests. The correlations were compared to those obtained for aBMD and for volumetric bone mineral density (vBMD) measured by computed tomography. The correlation of AaBMD to *F*_max_ adjusted for donor’s age was significantly higher than for aBMD and vBMD (*r* = 0.740, 0.658, and 0.609, respectively, *p* < 0.05). The differences between partial correlation coefficients for *P*_max_ to AaBMD, aBMD and vBMD relationships were smaller (*r* = 0.764, 0.720, and 0.732, respectively, *p* < 0.05), but also showed the superiority of AaBMD. Combining antero-posterior DXA aBMD and the lateral *h*_a_/*h*_p_ ratio, measured, for example, by the Vertebral Fracture Assessment software of the new generation of DXA devices, seems to accurately predict the mechanical vertebral parameters related to bone strength. It is assumed that the proposed AaBMD parameter may be more predictive for fracture risk assessment, which requires further studies.

## Introduction

The assessment of bone fracture risk (FR) is an important healthcare issue in developed countries, especially with regard to diagnosis and treatment of osteoporosis [1]. Fractures are associated with severe consequences, including long-term disability or death, as well as high healthcare costs [2]. Several methods are available for FR assessment and currently the FRAX model seems to be the most widely used. FRAX combines clinical risk factors and can include femoral neck areal bone mineral density (aBMD) to estimate 10-year fracture probability [1, 3, 4]. The only quantitative parameter involved in the FRAX calculator that directly describes bone tissue is aBMD measured by dual-energy X-ray absorptiometry (DXA) in the femoral neck [2, 3]. This is due to sufficient reference databases for this region of interest and high accessibility, as well as the relatively low costs of DXA equipment. A FRAX algorithm is still under development [1] and potential contribution of other quantitative bone strength (BS) descriptors in the future cannot be excluded.

aBMD measurements at different regions are not well correlated [5] and so the prediction of FR in one region on the basis of a measurement in another region is problematic [6]. A question, therefore, is whether the introduction of additional or different quantitative parameters into FRAX, e.g., the lumbar spine aBMD, would improve the FR prediction. Investigators are currently searching for novel parameters related to FR [7–9].

FR can be defined as the ratio between the load under particular loading conditions and the ultimate load supported by the bone, which is related to BS [9–12]. BS cannot be measured in vivo directly so parameters related to BS are of great interest. Even though FR assessment is currently based on the measurement of aBMD, some authors have shown that it is not an ideal tool for the correct assessment of FR [13].

It is believed that aBMD is only a surrogate marker of BS, and other determinants should be taken into consideration, in particular trabecular bone micro- and macro-architecture [13], as well as bone dimensions [14] and shape [12]. Some studies provided data showing an independent role for the vertebral dimensions in compressive strength and showed that small vertebrae with a reduced cross-sectional area demonstrate higher FR [13, 15–18].

Computed tomography (CT) is an alternative to DXA and is currently the only available technique that allows for estimation of true volumetric bone mineral density (vBMD). The vBMD measured in CT appears to be the best FR and BS predictor due to the fact that, in contrast to DXA aBMD, it is not affected by body or skeletal size, and the properties of surrounding tissues [19, 20]. Several studies directly comparing CT results to parameters derived from DXA characterizing BS in vertebral bodies showed similar correlations to FR. There are also numerous papers reporting CT superiority in this issue [21]. Our previously published results [22] confirmed that CT is the best predictor for BS.

DXA measures the areal density (not the volumetric density) corresponding to the ratio between bone mineral content and the area of scanned bone. The relatively high predictability of BS by aBMD could be partially explained by the fact that bone size is indirectly involved in DXA measurements [14].

The increase of bone fragility with age is attributed primarily to bone density loss; however, changes of bone geometry may also influence BS [16]. Therefore, vertebral size should be considered as a potential independent vertebral FR factor [17]. An enlargement of the external bone diameter with age is the effect of periosteal bone apposition and endosteal resorption with thinning of the cortex [11]. This is probably a mechanism to compensate for the decreased bone mass and the alterations in trabecular architecture [14, 16, 18]. Also, long-term bearing activity can result in an increase of the external diameter of bones supporting the load [16].

The idea of using geometrical parameters in FR prediction is not new. Supplementary geometrical parameters were applied for the antero-posterior (AP) aBMD aiming at the improvement of FR prediction [19, 22–25]. Several authors have proposed to utilize certain geometrical parameters as independent variables related to FR [13, 26].

Wren et al. [19] adjusted DXA aBMD by the AP bone area and height in order to estimate vBMD and volumetric bone mineral content (vBMC). vBMD and vBMC obtained by DXA results adjustment were subsequently compared to the results of CT. vBMC obtained from DXA and CT showed a significant correlation (*r* ^2^ = 0.94), while volumetric densities had a poorer correlation.

Lateral (LAT) heights or their ratios have been used for the assessment of prevalent or incident vertebral fractures [27, 28]. These supplementary measurements are of high clinical importance since vertebral fractures are the most prevalent osteoporotic fractures that should be taken into account, while tailoring osteoporosis management [28]. Low aBMD and past vertebral fractures are independent predictors of vertebral and non-vertebral FR [29]. The previous fractures or fractures in parents are taken into consideration in FRAX calculations [1, 4].

Diacinti et al. [13] proposed a new morphometric index, i.e., the sum of anterior vertebral body heights (AHs) from T4 to L5 for FR assessment in postmenopausal women. They proved that diagnostic accuracy of AHs was significantly higher when compared to that of lumbar spine aBMD and femoral neck aBMD.

Kolta et al. [18] noted a significant correlation between vertebral anterior heights (*h*_a_) and NTX/creatinine ratio, one of the key biochemical markers of bone resorption. They reported h_a_ reduction with advancing age in postmenopausal women, but did not observe any significant change in premenopausal women.

Measurements of vertebral aBMD are usually limited to AP projections. However, in this scanning modality, the results could possibly be influenced by the posterior vertebrae elements, aortic calcifications, and osteoarthritis of the spine seen in the majority of elderly patients [30]. These confounding elements can be excluded or reduced when using LAT scanning. A number of studies using estimation of aBMD based on LAT DXA have shown a stronger relationship with vertebral fracture frequency, ultimate load, or the age of patients compared to aBMD from AP projections [30].

The possibility of combining both the AP and LAT DXA results was also tested [7, 8, 13], giving promising results. The predictions based on paired AP and LAT DXA scans have a higher value than those done on the basis of AP scans alone [24].

Vertebral body heights were used in the assessment of vertebral FR [13] and in the assessment of vertebral fractures [27, 28]. The results suggested that LAT heights contain the relevant information connected to the vertebral BS. This is probably because they are affected by previous vertebral fractures, the patient’s posture, and loads supported by the vertebrae.

The aim of this research was to examine whether adjustment of the aBMD, measured using AP DXA, by the ratio of LAT anterior and posterior heights improves DXA utility in BS assessment.

## Materials and Methods

**Fig. 1:**
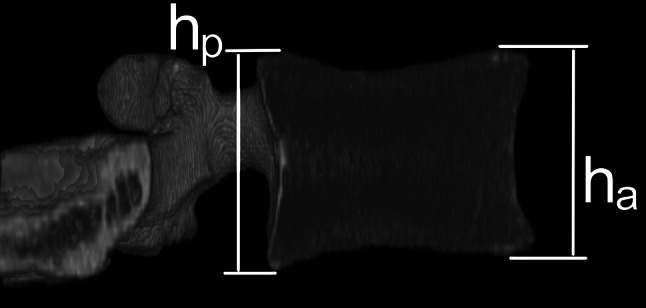
The lateral cross-section through the reconstructed three-dimensional image of a vertebra considered in the study. The method of measuring anterior and posterior heights is presented

A new parameter for indirect vertebral BS assessment is introduced. The result of the AP DXA measurement of the lumbar spine (aBMD) is adjusted by the results of geometrical vertebrae measurement in the LAT view. The ratio of anterior vertebral body height (h_a_) to posterior vertebral body height (h_p_) (Fig. 1) defines a novel adjusted areal bone mineral density (AaBMD):

1 $${\text{AaBMD}} = \frac{\text{aBMD}}{{(h_{a} /h_{p} )}}.$$

This study was approved by the local bioethics committee. Thirty-one cadaveric L3 vertebrae were examined. Vertebrae were collected from males, aged 22–81 years (mean 54, SD 19). Our study aimed to assess BS (and potentially FR) in patients who had not been diagnosed with vertebral fractures. The mechanical vertebrae properties are certainly influenced by past fractures and so the geometrical measurements and a semi-quantitative method of the vertebrae fracture assessment [2, 28] were used to eliminate past vertebral fractures. In order to reduce the potential influence of cadaver preservation on bone mechanical properties, all measurements were performed within 4 days after skeletal material was acquired. Samples were embedded in plastic containers (20 cm in diameter, 12 cm in height) filled with 0.9 % NaCl solution to simulate soft tissue and subsequently investigated with CT and DXA.

In previous research [22], we established that vBMD measured using CT is the best predictor for BS, and therefore, we used CT in the present protocol as the reference modality to compare the results of the novel AaBMD and the traditionally used DXA aBMD.

A Siemens Somatom Sensation 10 (Siemens, München, Germany) CT unit was used for CT measurements. Applied scanning parameters were as follows: X-ray tube voltage 120 kVp; exposition 120 mAs; slice thickness 0.6 mm; field of view 75 mm; image size 512 × 512 pixels. Images were reconstructed using an ultra-sharp reconstruction kernel.

It is believed that the properties of trabecular bone play a more significant role than the properties of cortical bone when considering vertebral mechanical strength [8, 14, 24], and so vBMD was measured only for the trabecular bone region within vertebral bodies. The Siemens Osteo-CT procedure was used for vBMD studies. The procedure is based on the comparison between the average Hounsfield Unit (HU) of the region of interest and the Siemens density standards.

A Lunar DPX-IQ (Lunar, Madison, US) densitometer was utilized for AP DXA measurements. The standard procedures for human AP bone density acquisition and analysis according to the manufacturer’s instructions were followed.

Geometrical measurements necessary for the ultimate stress calculation and for aBMD adjustment were performed on the reconstructed three-dimensional CT images. DICOM data acquired in CT studies were imported and reconstructed by custom-developed software based on the OpenGL library. The minimal axial cross-sectional area (*A*) of the vertebral body and both *h*_a_ and *h*_p_ vertebral body heights in the LAT view were estimated (Fig. 1).

Mechanical vertebrae properties were tested by an Instron 5566 testing device (Instron, High Wycombe, UK). Samples were prepared as described in our earlier paper [22]. Briefly, after the vertebrae posterior elements were removed, two layers of acrylic resin were placed on the top and bottom endplates of the vertebrae. Mechanical tests started with ten cycles of preloading and subsequently, displacement–load curves were collected. The ultimate load (*F*_max_) was extracted from the displacement–load curves, and the ultimate stress (*P*_max_) was calculated as described previously [9–11, 22] as the ratio between *F*_max_ and *A*:

2 $$P_{\hbox{max} } = \frac{{F_{\hbox{max} } }}{A}.$$

*F*_max_ and *P*_max_ as potentially the best estimators [10] of BS were correlated to aBMD, vBMD, and AaBMD by Pearson’s correlation. Due to a wide vertebrae donor’s age range, the partial correlations were calculated for age adjustment. Correlation coefficients were compared to evaluate the efficacy of the considered parameters as predictors of BS and FR. The statistical significance of correlation coefficient differences was tested using the means of the William’s formula and the procedure proposed by Steiger [31]. Differences were tested at a significance level of *p* = 0.05.

## Results

The results of parameters measured for all samples are presented in Table 1. Individual results of *F*_max_, *P*_max_, vBMD, aBMD, and AaBMD are presented, as well as the values of *h*_a_/*h*_p_ and donor’s age. Additionally, the change of aBMD caused by the *h*_a_/*h*_p_ adjustment was calculated (Δ = (aBMD − AaBMD)/aBMD*100 %) and placed in the last table column. The negative value of Δ means that AaBMD is higher when comparing to the original aBMD, while the positive Δ denotes cases in which the adjustment decreases aBMD. The adjustment ranged between −10.0 and 14.5 %. If recalculating the direct aBMD values into *T* scores, the observed adjustment would account for about ±(1/1.5) SD, which is a significant change in the context of osteoporosis evaluation.

**Table 1:** The most relevant results for samples involved in the study

Age (year)	F_max_ (kN)	P_max_ (MPa)	*h*_a_/*h*_p_	vBMD (g/cm^3^)	aBMD (g/cm^2^)	AaBMD (g/cm^2^)	Δ (%)
22	15.0	16.0	0.987	0.120	1.063	1.077	1.3
24	20.0	14.8	1.015	0.160	1.257	1.238	−1.5
25	19.4	14.9	1.007	0.124	1.166	1.158	−0.7
25	16.4	13.0	1.024	0.118	0.968	0.945	−2.3
30	19.9	18.4	1.032	0.154	1.475	1.429	−3.1
34	11.5	11.0	1.028	0.134	1.001	0.974	−2.7
40	14.6	10.5	0.997	0.101	1.012	1.015	0.3
40	10.0	8.6	0.983	0.080	0.787	0.800	1.7
40	18.1	15.9	0.941	0.106	0.965	1.026	6.3
41	15.1	11.8	0.997	0.112	1.001	1.004	0.3
41	16.2	14.8	1.068	0.169	1.527	1.430	−6.3
50	12.5	8.5	0.990	0.084	0.81	0.818	1.0
52	18.3	14.2	1.037	0.130	1.336	1.289	−3.5
52	11.7	10.2	1.036	0.062	0.906	0.875	−3.5
52	18.3	14.8	1.099	0.144	1.313	1.195	−9.0
53	9.8	8.9	1.104	0.073	0.843	0.764	−9.4
63	9.8	8.3	1.027	0.064	0.889	0.866	−2.6
64	10.5	6.0	1.053	0.034	0.916	0.870	−5.1
65	12.2	8.8	0.926	0.063	0.78	0.843	8.0
66	12.7	10.0	0.959	0.082	0.721	0.752	4.3
66	12.4	7.8	0.874	0.029	0.837	0.958	14.5
66	10.2	6.5	1.041	0.042	0.852	0.819	−3.9
69	8.2	5.5	1.033	0.026	0.553	0.535	−3.2
69	7.2	5.1	1.076	0.031	0.756	0.703	−7.1
70	9.5	8.7	1.059	0.074	0.761	0.719	−5.5
70	9.5	6.8	0.959	0.026	0.777	0.810	4.3
71	5.6	5.5	1.027	0.042	0.893	0.869	−2.6
75	16.2	11.0	0.983	0.078	1.07	1.089	1.7
79	6.7	4.5	1.055	0.033	0.747	0.708	−5.2
81	6.9	4.7	1.111	0.044	0.707	0.636	−10.0
81	4.5	3.4	1.055	0.027	0.664	0.630	−5.2
54 ± 19	12.5 ± 4.5	10.0 ± 4.1	1.02 ± 0.06	0.08 ± 0.05	0.95 ± 0.24	0.93 ± 0.23	−1.6 ± 5.3

*F*_*max*_ ultimate load, *P*_*max*_ ultimate stress, *h*_*a*_ */h*_*p*_ posterior to anterior vertebral body heights ratio measured in lateral view, *vBMD* volumetric bone mineral density obtained from CT, *aBMD* antero-posterior areal bone mineral density measured in DXA, *AaBMD* areal bone mineral density adjusted by *h*_a_/*h*_p_, *Δ* the areal bone mineral density change caused by the *h*_*a*_/*h*_p_ adjustment, i.e., Δ = (aBMD − AaBMD)/aBMD*100 %. The last row contains the average values and the standard deviations

Both *F*_max_ and *P*_max_ used as BS descriptors were considered and correlated to vBMD, aBMD, and the newly introduced AaBMD. The results of Pearson’s correlation are presented in Table 2 for *F*_max_ and *P*_max_. The influence of the wide range of donors’ ages was considered by calculations of partial correlations. The results of partial correlations are also presented in Table 2. All calculated correlation coefficients revealed statistically significant linear correlations with both *F*_max_ and *P*_max_ (*p* < 0.05). AaBMD seems to be the best predictor for *F*_max_ as well as for *P*_max_ due to the highest correlation coefficients after the age adjustment. The Hostelling test applied for correlation coefficients comparison proved the statistical significance of the observed differences.

**Table 2:** The correlation coefficients for the dependencies between vertebral ultimate load (*F*_max_), ultimate stress (*P*_max_), and considered indices: volumetric bone mineral density (vBMD) measured using CT, antero-posterior aBMD measured using DXA and areal bone mineral density adjusted by the *h*_a_/*h*_p_ ratio (AaBMD) (*h*_a_—anterior vertebral body height, *h*_p_—posterior vertebral body height)

	Correlation with *F*_max_		Correlation with *P*_max_	
	Pearson’s	Age-adjusted	Pearson’s	Age-adjusted
vBMD	0.843	0.609	0.912	0.732
aBMD	0.809	0.658	0.835	0.720
AaBMD	0.862	0.740	0.869	0.764

*p* < 0.05 for all cases

Correlations between *h*_a_/*h*_p_ and *F*_max_, *P*_max_, age as well as aBMD were also calculated, but no statistical significance was found in these cases (Table 3).

**Table 3:** The correlation coefficients (*r*) and confidence levels (*p*) describing relationships of *h*_a_/*h*_p_ ratio to *F*_max_, *P*_max_, age, aBMD, and vBMD (h_a_—anterior vertebral body height, h_p_—posterior vertebral body height)

Correlation to:	*r*	*P*
*F*_max_	−0.236	0.201
*P*_max_	−0.146	0.433
Age	0.134	0.475
aBMD	0.118	0.526
vBMD	0.062	0.788

**Fig. 2:**
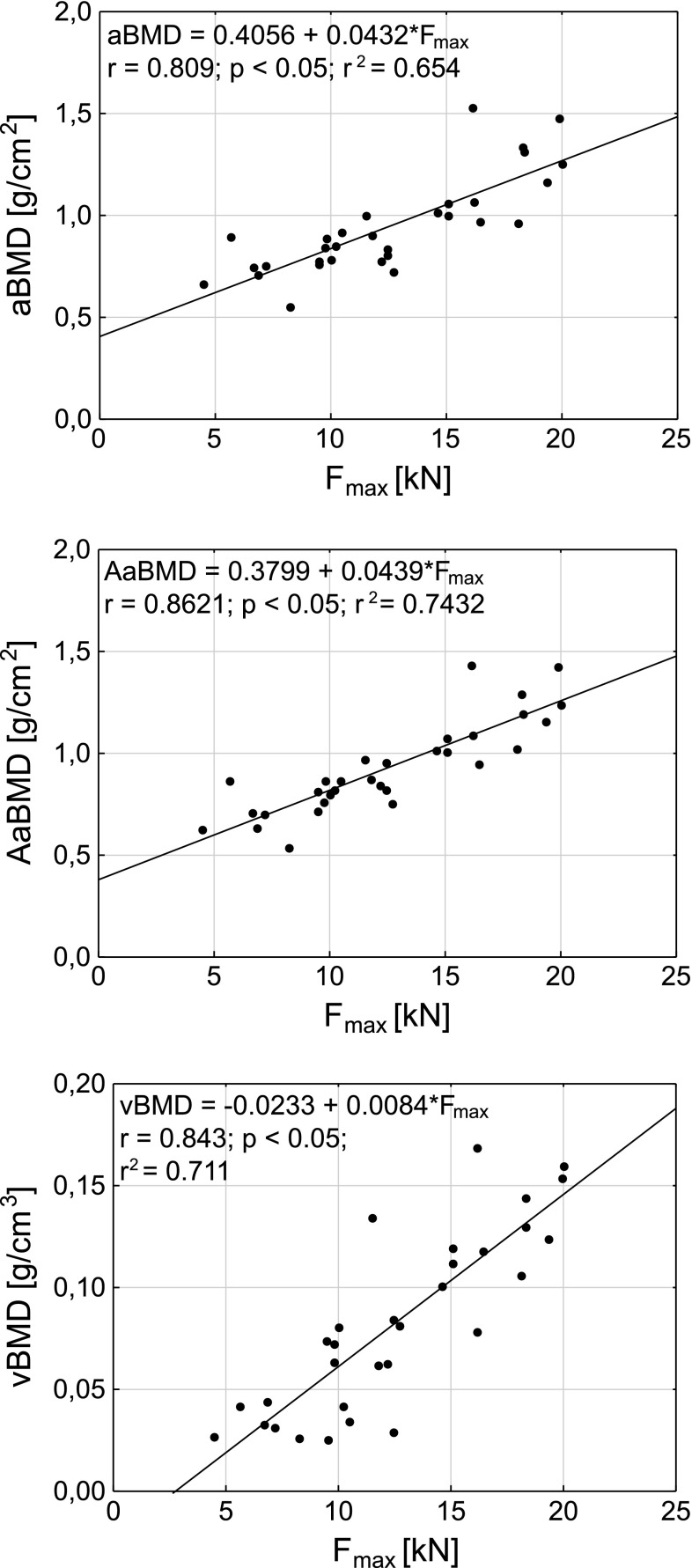
The dependencies between vertebral ultimate load (*F*_max_) and densitometric parameters. *aBMD* antero-poterior DXA areal bone mineral density, *AaBMD* aBMD adjusted by the anterior to posterior vertebrae heights ratio, *vBMD* volumetric bone mineral density

**Fig. 3:**
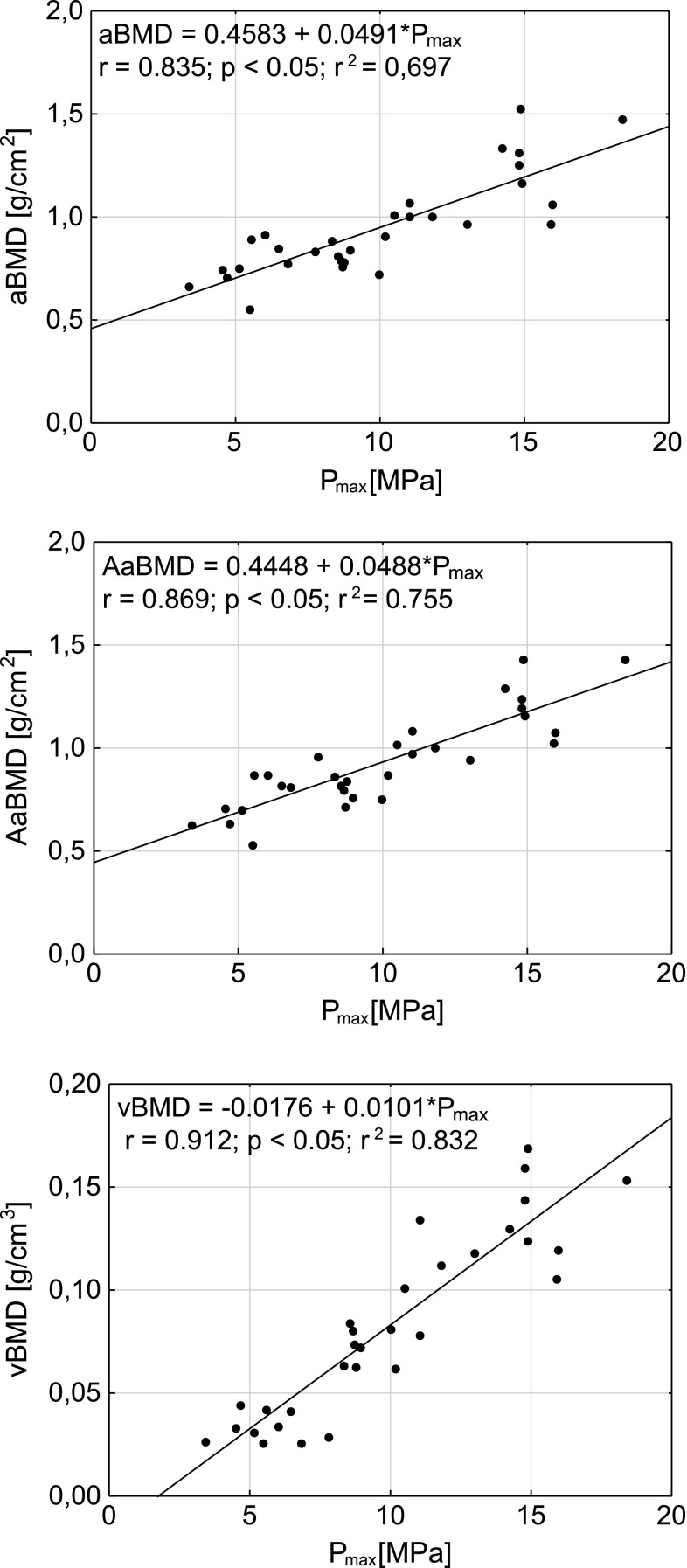
The dependencies between vertebral ultimate stress (*P*_max_) and densitometric parameters. *aBMD* antero-poterior DXA areal bone mineral density, *AaBMD* aBMD adjusted by the anterior to posterior vertebrae heights ratio, *vBMD* volumetric bone mineral density

The plots of vBMD, aBMD, and AaBMD as functions of *F*_max_ and *P*_max_ are presented in Figs. 2 and 3, respectively. All dependencies are shown with the best-fitting linear functions.

## Discussion

We propose the innovative parameter, AaBMD, to describe BS and to have potential utility in the management of osteoporosis. The correlations of AaBMD with *P*_max_ and *F*_max_ are greater than for the commonly used and widely accepted aBMD obtained using DXA scanning. The correlation is greater both before and after age adjustment. This leads us to the conclusion that AaBMD predicts the mechanical properties of the vertebrae more accurately and would probably also work better as the FR predictor.

Several authors believe that vBMD measured by CT is the most reliable parameter in assessing BS and FR due to the fact that CT measurement is not affected by body or skeletal size and properties of the surrounding tissues [19, 20]. For the same reason, others claim that *P*_max_ is a more reliable parameter than *F*_max_ when considering the mechanical properties of BS [9, 32]. Even though *P*_max_ contains adjustments for body and skeletal size, many authors still use *F*_max_ in their studies, or both *P*_max_ and *F*_max_ [9, 10, 20, 32]. In our research, we analyzed both parameters mentioned above.

The measurement of vBMD for quantifiable bone quality was a key procedure in our study. We found that AaBMD correlated better with *F*_max_ than with vBMD. The relationships with *P*_max_ were not as evident. Direct calculation of Person’s coefficient suggests superiority of vBMD over AaBMD (0.912 vs. 0.869) but, after the adjustment for age, the relationship reverses. The partial correlation calculated for the *P*_max_ versus vBMD relationship accounts for 0.732, while that for *P*_max_ versus AaBMD is higher (0.764). The difference is not large, yet, is statistically significant, which was confirmed by employing the procedure proposed by Steiger [31].

Our results strongly suggest that AaBMD is better than vBMD in predicting vertebrae mechanical strength. The reason why aBMD adjustment by *h*_a_/*h*_p_ ratio improves vertebral strength prediction is not known and needs further research.

The shape of vertebrae, reflected by h_a_/h_p_, can be the effect of cumulated previous microfractures of trabecular bone or the effect of bone adaptation to the physical loads [12, 33]. In addition to these two, also a natural external vertebrae sizes increase caused by the periosteal bone apposition influence the DXA accuracy [13, 14, 16]. Precision of DXA spine aBMD measurements is 1.0–1.5 %, while the assessed accuracy is 5–7 % [3]. The accuracy-related errors are mainly caused by the nonpredictable soft tissue amount, composition, and geometry, as well as unknown bone dimensions. The accuracy of 5–7 % for aBMD can be recalculated into ±0.5 SD in the *T* score, which is widely accepted as the diagnostic parameter in bone densitometry. Such low accuracy can change the diagnosis from nonosteoporotic into osteoporotic or vice versa and influence patient management.

Vertebral dimensions change considerably with age [16]. These changes are greater in older patients, and consequently, the corrections are more relevant for older individuals, a finding that was also supported by our results. Comparing aBMD to AaBMD (Table 1), a correction greater than the level of AP DXA accuracy (|Δ| > 5 %) [3] was observed for 2 out of 12 patients younger than 52 years (16.7 %). Such large corrections in the group of older individuals (≥52 years) were observed in 10 cases out of 19 (52.6 %).

The reasons why and when the aBMD adjustment by *h*_a_/*h*_p_ improves the correlation with *F*_max_ and *P*_max_ are not evident. The ratio of *h*_a_/*h*_p_ was not significantly correlated with *P*_max_, *F*_max_, aBMD, vBMD (Table 3), or other geometrical parameters of the vertebrae (results not presented).

The observed phenomena could be attributed to the changes in the effective attenuation coefficient of vertebrae caused by the inclined cortical endplates. As is shown in the Appendix, it is possible to calculate the correction to the linear attenuation coefficient caused by slight vertebral endplate inclination. After a few assumptions and simplifications, it can be shown that the correction is proportional to the *h*_a_/*h*_p_ ratio, and therefore, the correction to aBMD has to be proportional to this ratio. It should be pointed out that our conclusions concern natural vertebral shape being the effect of individual patient’s posture rather than the effect of previous vertebral fractures. The angle of cortical endplate inclination should be small and that is the case in our data. The average *h*_a_/*h*_p_ ratio is 1.02 ± 0.06 (Table 1). The maximal reduction of the shorter height (anterior or posterior) in relation to the longer is equal to 14.5 % in one case, 10 % in another, and less than 10 % in the remaining cases. The previous fractures, which could possibly lead to an artificial aBMD increase and a bone strength decrease, are accompanied by greater height reductions. According to Genant et al. [28], the vertebral deformity due to past fractures can be classified as mild when the reduction of any vertebral height is about 20–25 %.

The presented methodology, i.e., aBMD adjustment by *h*_a_/*h*_p_, is innovative, and to the best of the author’s knowledge is published for the first time. A few authors applied vertebral LAT heights for the assessment of vertebrae mechanical properties.

Ross et al. [34] investigated the relationship between vertebral body dimensions and FR in vivo. They analyzed *h*_a_/*h*_p_ as the parameter describing vertebral shape in the LAT view and found that a smaller *h*_a_/*h*_p_ ratio might increase fracture risk by shifting loads toward the anterior part of the vertebrae. They did not find a significant direct correlation between vertebral body heights or *h*_a_/*h*_p_ with FR, which is in agreement with our results, as we did not observe a statistically significant correlation between *h*_a_/*h*_p_ and *F*_max_ (*r* = −0.236, *p* = 0.201) or *P*_max_ (*r* = −0.146, *p* = 0.433).

The ratios of lateral heights *h*_a_/*h*_p_ and *h*_m_/*h*_p_ (*h*_m_: middle lateral vertebral body height) were considered by Sone et al. [26] as the parameters that allowed the assessment of mild vertebral fractures and the prediction of fracture susceptibility. They investigated T12–L4 vertebral height ratios of 479 pre- and postmenopausal Japanese females and the relationship of these ratios to age and aBMD. Height ratios, and especially *h*_m_/*h*_p_, tended to decrease with age and positively correlated with aBMD. The correlation coefficients were *r* = −0.125 (*p* < 0.05) for the dependence between *h*_a_/*h*_p_ and age for L3 vertebrae, while *r* = −0.180 (*p* < 0.01) for the relationship between age and *h*_m_/*h*_p_. Statistical analysis showed no significant correlation between aBMD and *h*_a_/*h*_p_ for L3 vertebrae, while *r* = 0.145 (*p* < 0.01) for the dependence between aBMD and *h*_m_/*h*_p_.

The most important conclusion in the Sone group report [26] was that the mean values of height ratios of nonfractured vertebrae were significantly lower in postmenopausal women with earlier vertebral fractures compared to the group without fractures. They concluded that the height ratios of nonfractured vertebrae are independent predictors of FR.

Our results do not allow us to draw a similar conclusion. There are some discrepancies: (1) we did not observe a statistically significant correlation between *h*_a_/*h*_p_ and aBMD; (2) Sone et al. calculated the correlation coefficient for the relationship between *h*_a_/*h*_p_ and age as negative, while this relationship in our study was positive; (3) there are no significant correlations between *h*_a_/*h*_p_ and *P*_max_ or between *h*_a_/*h*_p_ and *F*_max_. Both *P*_max_ and *F*_max_ describe directly the BS and have to be related to FR, so we conclude that *h*_a_/*h*_p_ cannot be an independent variable allowing FR prediction.

The discrepancies are probably due to the fact that the Sone group’s conclusions are based on a huge database (479 women), and they analyzed all vertebrae contained in the T12–L4 spinal region (≈2,200 samples). A huge sample number means that even if the correlation coefficient presents with a rather low value (*r* ~ 0.2), the confidence level stays below the accepted 0.05 value. This is not the case in our low number of samples. The order of magnitudes of correlation coefficients presented in Table 3 are the same as in the paper of Sone et al. [26], while the *p*-values are much higher than 0.05.

Another reason for the discrepancies could come from the imaging modality chosen for morphometric measurements. Our results come from CT cross-sections, while X-ray radiography was used in the Sone group’s research. The accuracy and precision of the LAT dimensions could be dependent on the imaging method applied. The same group, in another paper [27], reported that the values of *h*_a_ and *h*_p_ obtained from MRI sagittal cross-sections were larger than those obtained by X-ray morphometry, while the opposite relationship occurred when considering *h*_m_.

Another source of differences is the fact that the Sone group considered females, while we acquired data from males. Studies considering gender have indicated that vertebral sizes increase significantly in men with the age but there is a lack of consensus whether vertebral body expansion occurs in women [11]. We believe that the positive influence of *h*_a_/*h*_p_ adjustment on aBMD is partially the effect of considering the age-related changes. As our samples were from males, it is impossible to make any conclusions considering a female population on the basis of our data. This is one of the largest disadvantages of the presented work, and further investigations will address this issue.

Perilli et al. [30] investigated human vertebrae in vitro and compared results from AP DXA, LAT DXA, μCT, and mechanical tests. aBMD obtained in LAT projections demonstrated a significantly stronger relationship with structural information achieved using μCT in comparison to AP DXA. This suggests that vertebral aBMD and BMC measured using LAT DXA are most strongly related to the vertebral body bone volume and trabecular bone properties. Further, the correlations to ultimate load were stronger for LAT (*r* ^2^ = 0.70) than for AP aBMD (*r* ^2^ = 0.37) despite both being statistically significantly correlated. In conclusion, LAT DXA performed better than AP DXA when considering in vitro studies.

Despite promising results, LAT DXA projections cannot be easily applied in clinical practice for aBMD assessment. Conventional densitometry of LAT DXA scans is characterized by unacceptably high precision error (2.0–6.9 %) related mainly to the difficulties with repetitive patient positioning. This disadvantage can be eliminated by the use of C-arm-equipped densitometers, but some problems remain related to the LAT DXA procedure. Prospective studies should be undertaken to determine the predictive strength of LAT data compared to AP data for vertebral FR before the clinical utility can be established. Furthermore, the accuracy of in vivo LAT DXA is influenced not only by the patient positioning but also by the heterogeneous nature of soft tissue surrounding the spine and higher X-ray beam attenuation related to the greater soft tissue volume in LAT geometry compared to AP geometry [30]. On the other hand, Vertebral Fracture Assessment (VFA) software of the new generation of densitometers [35, 36] can be utilized for precise and reliable assessment of vertebral geometry in AP and LAT views of the thoracic and lumbar spine.

It is beyond doubt that AP projections are insufficient to describe three-dimensional objects like vertebrae and the additional information concerning the third dimension apart from the AP view would improve the vertebral FR prediction. Performing both, AP and LAT measurement, could be one solution, but this is not perfect due to the LAT DXA limitations [30]. Our approach is different, and utilizes the AP aBMD adjustment by geometrical data from the LAT view, and more precisely by *h*_a_/*h*_p_.

To make our application useful in the clinical setting, every AP aBMD assessment should be accompanied by assessment of vertebral heights in the LAT view. We assumed that CT allows the estimation of the vertebrae dimensions approaching the real values, and showed that the aBMD adjustment by the true heights improves the strength prediction. Our conclusions are based on CT geometrical measurements and, if considering precision, it would also represent the most accurate approach in clinical practice. On the other hand, it should be used with caution due to the high X-ray dose in spinal CT studies and higher costs. Despite standard radiography of the thoracic and lumbar spine still being the reference method for detecting vertebral fractures and LAT spine geometry assessment [36], a novel approach utilizing VFA should be considered instead for *h*_a_/*h*_p_ measurements. VFA seems to be superior in comparison to radiography because of considerably lower radiation exposure for the patient and a lack of image distortion. Another advantage is the possibility of performing AP DXA and LAT VFA in the same study when modern DXA equipment is utilized. We did not check whether the adjustment of aBMD by VFA results would be feasible in clinical setting as this requires further studies.

It should be pointed out that our conclusions are based on the L3 vertebrae only. Nevertheless, we believe that the same method could be used for other vertebra analyses. The potential use of the proposed method in other bones needs further research.

## Conclusions

We present a novel approach for the better evaluation of mechanical properties of vertebral bodies. This innovative index combines antero-posterior DXA measurement of aBMD with anterior and posterior vertebrae body heights. The original index is defined as the ratio of aBMD and *h*_a_/*h*_p_ (AaBMD = aBMD/(*h*_a_/*h*_p_)). AaBMD was correlated with vertebral ultimate stress and load measured in mechanical crush tests in vitro. The correlation between AaBMD and vertebral strength was stronger than correlations between DXA aBMD or CT volumetric bone mineral density and vertebral strength.

Therefore, AaBMD might be a better predictor of vertebrae strength than aBMD and vBMD. Moreover, this novel index might also work better as a predictor of fracture risk. Since fracture risk assessment is a challenging issue influenced by several factors discussed above, the relationship between the proposed AaBMD and FR requires further study.

## Figures and Tables

**Fig. 4:**
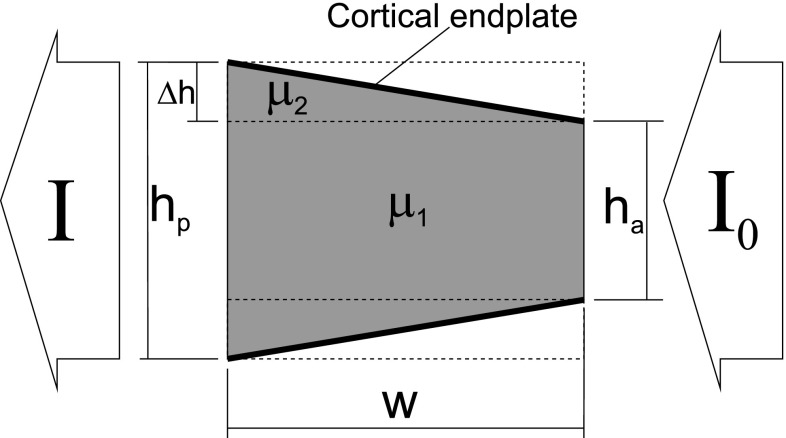
The scheme of the DXA study in the case of inclined vertebrae. The cortical endplate thinness and the inclination magnitude are exaggerated for scheme clarity. *I*_*0*_ incident beam intensity, *I* the intensity of X-rays passed through the vertebrae, *h*_*a*_ *and h*_*p*_ anterior and posterior vertebrae heights, *Δh* half of the difference between *h*_p_ and *h*_a_, *μ*_*1*_ the effective average linear attenuation coefficient of the vertebra body, *μ*_*2*_ the effective average attenuation coefficient of the inclined endplates regions

## Appendix

In DXA studies, the homogeneity of the vertebral body has been assumed and this may not be valid in practice when the vertebral endplates are inclined. Figure 4 shows a layer of a vertebral body (lateral cross-section, *w*_AP_ thickness) containing three areas characterized by *μ*_1_ and *μ*_2_ linear attenuation coefficients. The inclined cortical endplates change the attenuation in areas denoted by index “2” in comparison to the vertebral body attenuation *μ*_1_.

The effective linear attenuation coefficient *μ* will be given by the following expression:

3 $$\mu = \frac{1}{w}\ln \left( {\frac{{I_{0} }}{I}} \right),$$

where *I* can be expressed as

4 $$I = \frac{{I_{0} w_{\text{AP}} h_{\text{a}} }}{{w_{\text{AP}} h_{\text{p}} }}e^{{ - \mu_{1} w}} + \frac{{2I_{0} w_{\text{AP}} \varDelta h}}{{w_{\text{AP}} h_{\text{p}} }}e^{{ - \mu_{2} w}}$$

and in consequence

5 $$I = \frac{{I_{0} }}{{h_{\text{p}} }}\left( {h_{\text{a}} e^{{ - \mu_{1} w}} + 2\varDelta he^{{ - \mu_{2} w}} } \right).$$

Therefore, *μ* is given by

6 $$\mu = \frac{1}{w}\ln \left( {\frac{{h_{\text{p}} }}{{h_{\text{a}} e^{{ - \mu_{1} w}} + 2\varDelta he^{{ - \mu_{2} w}} }}} \right).$$

When Δ*h* ≪ *h*_p_ and *μ*_2_ *w* < 1, Eq. (6) simplifies to

7 $$\mu = \mu_{1} \left( {1 + \left( {\frac{{h_{\text{p}} }}{{h_{\text{a}} }} - 1} \right)\left( {\frac{{\mu_{2} }}{{\mu_{1} }} - 1} \right)} \right).$$

If *μ*_2_/*μ*_1_ ≈ 2, we finally obtain

8 $$\mu = \mu_{1} \frac{{h_{p} }}{{h_{a} }}.$$

After assuming that the volumetric density of the whole vertebrae is not considerably altered by the inclined endplates, Eq. (8) can be rewritten as

10 $${\text{AaBMD}} = \frac{\text{aBMD}}{{{{h_{\text{a}} } \mathord{\left/ {\vphantom {{h_{\text{a}} } {h_{\text{p}} }}} \right. \kern-0pt} {h_{\text{p}} }}}},$$

which is exactly the same as Eq. (1).

The assumption that *μ*_2_/*μ*_1_ ≈ 2 is reliable if we consider the energy range used in DXA. The *μ*_2_/*μ*_1_ ratios were estimated considering that vBMD averaged over the whole vertebrae is about 0.3 g/cm^3^ [19] and by applying the data of the National Institute of Standards and Technologies (http://www.nist.gov/pml/data/xraycoef/index.cfm). The calculated ratios were 2.19, 1.97, and 1.73 for 50, 60, and 80 keV of X-ray energies, respectively.
